# Integrating biological pathways and genomic profiles with ChiBE 2

**DOI:** 10.1186/1471-2164-15-642

**Published:** 2014-08-03

**Authors:** Özgün Babur, Ugur Dogrusoz, Merve Çakır, Bülent Arman Aksoy, Nikolaus Schultz, Chris Sander, Emek Demir

**Affiliations:** Computational Biology Center, Memorial Sloan-Kettering Cancer Center, 1275 York Avenue, Box 460, New York, NY 10065 USA; Department of Computer Engineering, Bilkent University, Ankara, 06800 Turkey; Tri-Institutional Training Program in Computational Biology and Medicine, 1275 York Avenue, New York, NY 10065 USA

**Keywords:** Pathway informatics, Genomic data analysis

## Abstract

**Background:**

Dynamic visual exploration of detailed pathway information can help researchers digest and interpret complex mechanisms and genomic datasets.

**Results:**

ChiBE is a free, open-source software tool for visualizing, querying, and analyzing human biological pathways in BioPAX format. The recently released version 2 can search for neighborhoods, paths between molecules, and common regulators/targets of molecules, on large integrated cellular networks in the Pathway Commons database as well as in local BioPAX models. Resulting networks can be automatically laid out for visualization using a graphically rich, process-centric notation. Profiling data from the cBioPortal for Cancer Genomics and expression data from the Gene Expression Omnibus can be overlaid on these networks.

**Conclusions:**

ChiBE’s new capabilities are organized around a genomics-oriented workflow and offer a unique comprehensive pathway analysis solution for genomics researchers. The software is freely available at http://code.google.com/p/chibe.

## Background

A key challenge in genomics is to predict the phenotypic effects of genomic alterations and their combinations. Genomic alterations affect phenotypic changes via a complex interplay between multiple genes and their products [1, 2]. Decades of molecular biology research have elucidated a substantial portion of this network, which is currently being reconstructed at a high level of detail that captures cellular processes such as transcriptional regulation, post-translational modification, transport, and complex formation in the formal and computable BioPAX format [3]. The corpus of publicly available biological pathway data in the BioPAX format is continuously expanding, both in terms of cellular processes coverage and the level of curation detail [4]. These computable models of cellular processes can substantially improve high-throughput data analysis by linking correlation to causation. The current landscape of high-throughput profile analysis, however, is dominated by correlation-based methods that are either *ab initio* or that use gross simplifications of biological processes [5–9], which can only capture relatively straightforward associations. There are three equally important technical challenges in making rich pathway information more accessible to researchers: (i) finding a subset of the aggregate corpus that is relevant to the biological problem at hand; (ii) presenting this network of interest in an intuitive manner, properly reducing complexity, and still allowing exploration at multiple levels of detail; and (iii) mapping high-throughput data on top of networks of interest for analysis.

**Figure 1:**
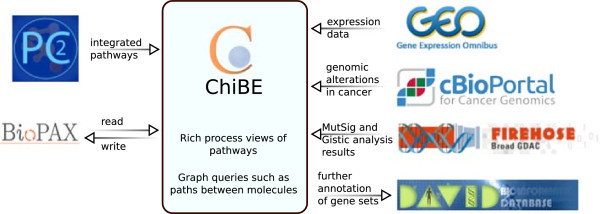
**ChiBE data flow.** Diagram showing the data flow between ChiBE and related resources.

Chisio BioPAX Editor (ChiBE) [10] visualizes BioPAX models as detailed process diagrams, displaying molecules, complexes, reactions, and cellular compartments. We developed version 2 of ChiBE with the above goals in mind to help scientists formulate and answer biological questions by combining rich pathway information with genomic and expression profiles. Users can use local models or obtain them from the Pathway Commons [11] database using advanced graph searches (neighborhoods, paths between molecules, or common targets/regulators) and overlay genomic alteration data from the cBioPortal for Cancer Genomics [12] or expression data from the Gene Expression Omnibus (GEO) [13] (Figure 1). These facilities are provided within the software tool, with no need for manual download or data handling.

In the following sections, as a general demonstration of ChiBE, we explore two example biological questions and generate hypotheses using ChiBE. We then summarize the capabilities of ChiBE version 2 and discuss how it compares to other pathway visualization tools. For more detailed instructions on how to use each of the available features, please refer to the ChiBE User’s Guide ( http://www.cs.bilkent.edu.tr/~ivis/chibe/ChiBE-2.2.UG.pdf).

## Implementation

ChiBE is implemented in Java and built on top of ChiEd 2.0, a generic graph visualization tool based on the Eclipse Graph Editing Framework 3.1.1. ChiBE uses Paxtools [14] for handling BioPAX data, and uses PATIKA*mad* [15] for handling and mapping profiling data. ChiBE was designed to be easily extensible programmatically to create custom toolbars, tooltips, and context menus to enable domain-specific customization.

## Results and discussion

### Exploring biological pathways with ChiBE

#### ChiBE complements and improves existing pathway analysis in endometrial carcinoma

**Figure 2:**
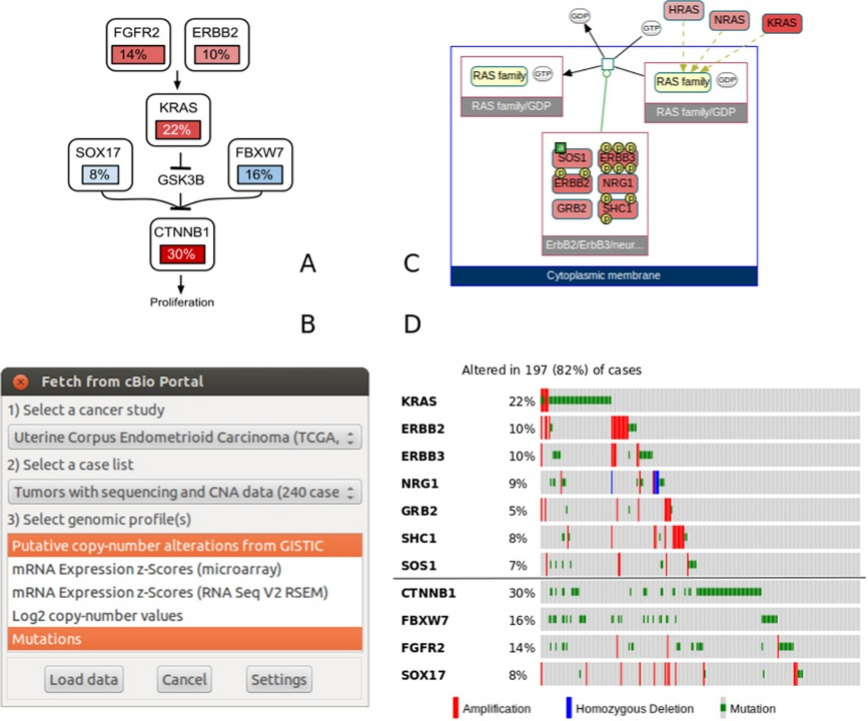
**Steps in sample use case 1. A)** A reconstruction of the pathway published in the manuscript by the TCGA network [16]. In the original figure, alteration frequencies are shown for three subgroups of cases, but we merge these into one in this version. **B)** Dialog to retrieve alteration data from the cBioPortal. **C)** Paths from ERBB2 to KRAS in Pathway Commons, overlayed with alteration data of endometrial cancer from the cBioPortal. This diagram is part of the result that was automatically generated by ChiBE using the menu item "Query | Pathway Commons (Level 3) | Paths From To". **D)** Oncoprint of the related genes, generated by the cBioPortal. Genes from C are shown above the black line, and altered genes from A are shown under the black line.

A recently published The Cancer Genome Atlas (TCGA) article [16] reports genomic, transcriptomic and proteomic profiling of 240 endometrial tumors. In the *pathway analysis* section, the authors present a network, discovered by searching for mutually exclusive alteration patterns (Figure 2A). This network of binary signal transduction is a useful but simplified model of the true biology; signaling between two proteins is generally much more complex. To explore the details of the signaling events in this figure, we start by searching for paths of length 1 from ERBB2 to KRAS in Pathway Commons, using ChiBE. This search returns one reaction that captures the transfer of GTP to the RAS family of proteins by a complex containing ERBB2. We then overlay mutation and copy number alteration data from the TCGA endometrial cancer dataset from the cBioPortal using the portal data import dialog (Figure 2B). Upon loading the data, gene alteration frequencies are color coded on the related molecules in the graph. The resulting diagram (Figure 2C) reveals that not only ERBB2, but all members of the complex have substantial alterations. ERBB3, for instance, has the same frequency of alterations (10%) as ERBB2. None of the members of this complex other than ERBB2 is mentioned in the TCGA article; however, at least one member of this complex is altered in 35% of patient samples (Figure 2D). Adding KRAS to this set increases the fraction of altered samples to 49%. This example clearly demonstrates that we can reproduce and improve existing literature findings with ChiBE.

#### ChiBE identifies an altered complex in breast cancer

In this example, we start with a list of frequently mutated genes in breast cancer. Such a list can be obtained using the MutSig tool [17] provided by the cBioPortal. With ChiBE, we run a "paths-between" query on the Pathway Commons database using the top 15 mutated genes (PIK3CA, TP53, MAP3K1, KMT2C, GATA3, CDH1, MAP2K4, TBX3, RUNX1, PTEN, PIK3R1, AKT1, CTCF, NCOR1, and RPGR) as the query seed with a default path length limit of 1. This query will find the linkage between these entities and will return them in BioPAX format. The result is then automatically laid out using the CoSE compound spring embedder algorithm [18] and displayed as a detailed process diagram.

Because the seed proteins of interest in the query are central in the cell, the query returns a large network, which is hard to understand visually. To reduce the complexity, ChiBE automatically hides subcellular compartments for large networks in the view. This view highlights all the seed genes and shows the connections between them.

**Figure 3:**
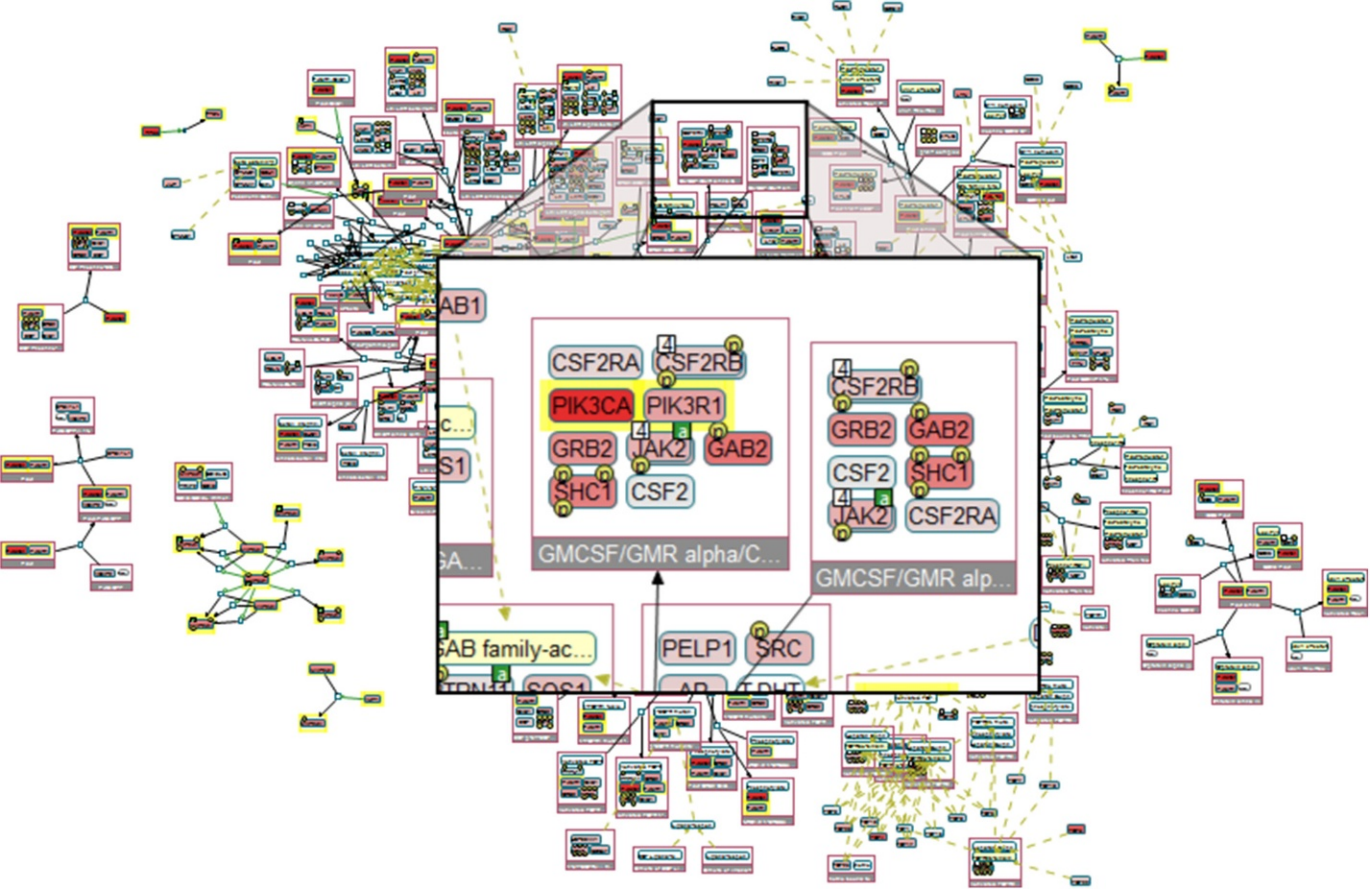
**Paths between frequently mutated genes.** The paths-between query result, with alteration data overlaid.

**Figure 4:**
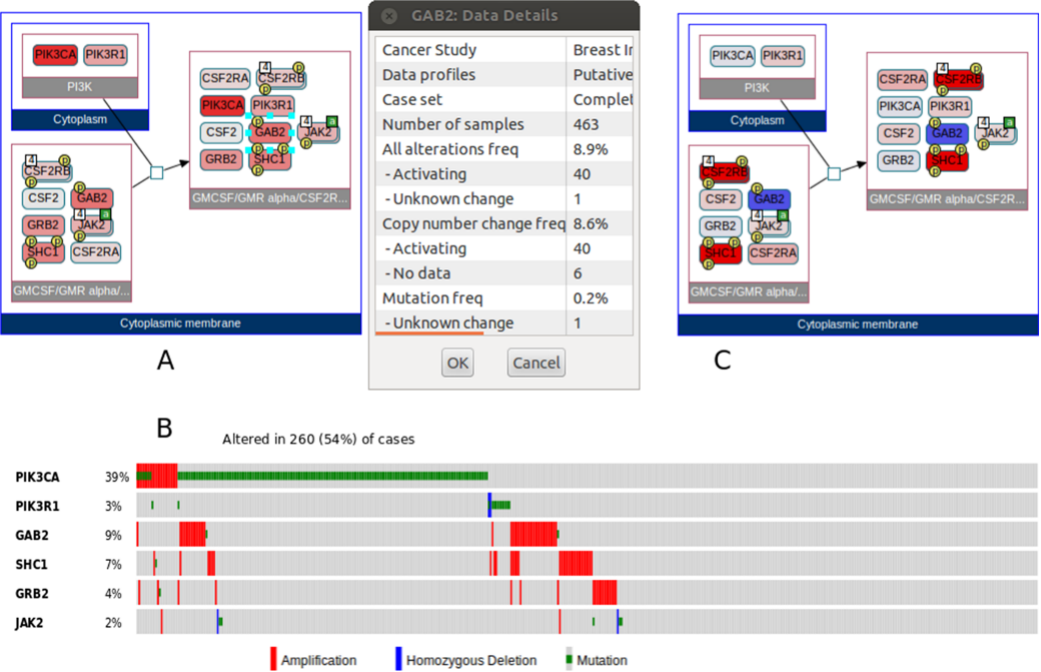
**Steps of sample use case 2. A)** The reaction of interest in a separate view displaying compartments and detailed properties of GAB2. **B)** Oncoprint of altered genes, provided by the cBioPortal. **C)** The same reaction but this time expression data is overlaid on involved molecules.

We then overlay mutation and copy number variation data from the cBioPortal of the study "Breast Invasive Carcinoma (TCGA, Nature 2012)", and generate the graph shown in Figure 3. We notice that some of the complexes contain frequently mutated (seed) genes, as well as other genes with some significant alteration. One of these genes is a complex containing JAK2. To focus on this particular complex, we run a local neighborhood query from the pop-up menu. The resulting view shows that the PIK3CA/PIK3R1 complex in the cytoplasm binds to a large complex of active JAK2 on the cytoplasmic membrane. We can obtain details of the alterations using the pop-up menu for each molecule (Figure 4A). We observe that GAB2, SHC1, and GRB2 are mostly copy number amplified in breast cancer patients. Alterations concentrating around a common function suggests that altering this function can be a driver event for the cancer.

We further investigate this hypothesis by looking for differential expression of members of this complex in datasets submitted to GEO. Searching GEO datasets with the keywords "tumorigenic and normal breast" returns a dataset by Liu *et al.* [19]. Within ChiBE, the user can automatically download this dataset by providing its GEO series ID of GSE6883. After tagging normal and disease samples using the data management dialog, node colors are updated to code the fold changes between average values of normal and disease samples (Figure 4C), and fold-change values are displayed with tooltips. We observe that SHC1 and CSF2RB are over-expressed in tumorigenic samples more than twofold (2.21 and 2.56, respectively), and that GAB2 is under-expressed 1.65-fold relative to normal samples. Thus, the expression dataset also supports the idea that this complex can have some significant function in tumorigenic behavior of breast cancer cells. We can continue this study by performing new queries, for instance, to retrieve the downstream paths from this complex or its members.

The above scenario shows the benefit of integrating rich pathway data with profiling data for exploring the biology of a disease. ChiBE automatically downloaded, formatted, and parsed the data. By removing these so-called "micro barriers", ChiBE offers efficient access to rich pathway information for high-throughput data analysis.

### Getting and working on a model

ChiBE was designed to work with pathways represented in the BioPAX format [3] - a community developed standard - including the latest version, Level 3. We call the entire set of biological information loaded from a BioPAX file (an OWL file) or obtained through a query into the Pathway Commons database, a *pathway model*, or simply a *model*. Each model is potentially composed of one or more pathways and their subpathways. Pathway boundaries and hierarchical organization are defined in the BioPAX model by the original curator.

**Figure 5:**
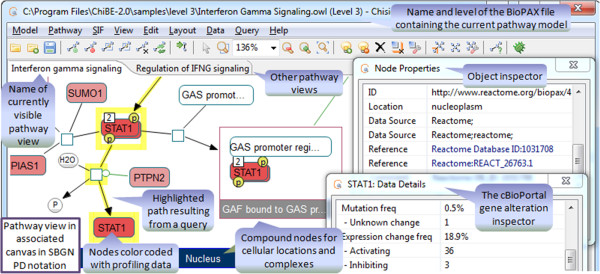
**ChiBE overview.** ChiBE views are organized in canvasses, each displaying one or more BioPAX pathways in a graphically rich, interactive manner. In this example, an Interferon Gamma Signaling pathway is displayed in process description notation.

ChiBE draws each pathway as a separate *view* (Figure 5). Users can close existing views or open new views from the underlying model. Views can also be manipulated graphically and saved with layout information. A model may also be expanded by merging with another BioPAX model (another OWL file or a Pathway Commons query), provided both models are at the same level of BioPAX. This option allows users to incrementally expand their pathway model by including other BioPAX models or queries.

The default ChiBE view uses a notation very similar to that of the SBGN Process Description (SBGN-PD) language [20]. Reaction nodes map to biochemical processes, while other nodes map to pools of molecules. Different states of the same "entity" (e.g., different phosphorylated forms of the same protein) are represented separately. Node labels and colors are used to indicate molecule pools that belong to the same entity. When profiling data is overlaid on pathways, however, node colors represent experimental values (as described later in the paper). ChiBE provides an array of interactive tools for the user to explore both types of views.

#### Retrieving a model from Pathway Commons

Pathway Commons [11] is a collection of publicly available pathway data from multiple organisms that enables biologists to browse and search a comprehensive collection of pathways from multiple sources represented in a common language. Using keywords of pathways or molecules, ChiBE can automatically query and obtain pathways from Pathway Commons through its web service. More importantly, ChiBE can also run "graph-queries" to find connections between entities, even when these connections span multiple pathways. Available graph-based queries that allow users to answer questions such as "Is there a known multi-step signaling path between Protein A and Protein B?" and "What is the network that connects these sets of altered genes?" are discussed in detail in the following section. One can also perform queries with keywords and identifiers.

After obtaining a network of interest, users also have the option to send the selected genes to DAVID bioinformatics service [21] for further gene-set related analysis.

#### Querying for enriched reactions

Identification of altered parts of an interaction network based on experimental data has been previously studied [22–24]. ChiBE adapts this approach to detailed networks by detecting reactions, whose list of participants are significantly enriched with altered genes, or other genes that are of interest to the user. The set of altered genes can be selected among recurrently mutated and/or copy number altered genes in a TCGA study, by providing the code of the study and significance thresholds for recurrence. ChiBE retrieves these genes from Broad Firehose.

### Graph queries

ChiBE provides several graph queries for retrieving specific portions of the cellular process network. ChiBE can query the Pathway Commons database using its web service interface. Alternatively, a query can be executed locally on the currently loaded model.

Remote graph queries to Pathway Commons include: neighborhood, paths-between, paths-from-to, and commonstream. These queries are based on the algorithms given in [25], with modifications to accommodate additional BioPAX semantics such as generic molecules. In each case, the user provides the query seed using HGNC gene symbols [26], a path length limit, and other query parameters. The result of the query is merged with the current model, allowing users to iteratively build a model using queries.

**Neighborhood:** This query retrieves the pathway neighborhood of the seed genes. Users can search upstream, downstream, or in both directions. By calling this query consecutively, users can easily explore pathways.

**Paths-between:** This query computes a connecting network between the seed genes. The connecting paths may include *linker* molecules up to a user-defined threshold. This query is especially useful for putting the results of high-throughput screens into the pathway context.

**Paths-from-to:** This query is similar to the paths-between query; the key difference is that this one takes two sets of seeds (source and target) and brings the merged network of all paths from a source to a target gene.

**Common-stream:** This query retrieves the network composed of all genes that are at the upstream and/or downstream of *all* seed genes. This query can be used to find master regulators (upstream) or common targets (downstream) that are signal integrators.

The queries discussed above can also be performed on the local model. However, instead of HGNC gene symbols, entities of the local model are used as the query seeds and the search is limited to the currently loaded model in memory.

### Loading profiling data

ChiBE can use three different resources for profiling data: the cBioPortal cancer genomic profiles, NCBI GEO expression profiles, or tab-delimited files. With the first two options, ChiBE automatically downloads and maps the data on the network. The last option allows one to use private or preliminary datasets. In that case, ChiBE asks for a mapping between references in the tab-delimited file and references on the network through a wizard, and converts the data into an internal format for later use.

#### Loading data from the cBioPortal

The cBioPortal for Cancer Genomics [12] is a repository of cancer genomics datasets with about 69 cancer studies and more than 17,000 tumor samples. ChiBE accesses the cBioPortal through its web service interface. Users choose a cancer study, a case list, and the available genomic profiles to load (Figure 2B). Case lists are subsets of cases with some common properties (predefined in the cBioPortal) such as *all sequenced tumors*. ChiBE discretizes this data into alterations, and color codes gene alteration ratios on the network (see Figure 2C for an example). For continuous valued experiments, users can set thresholds for discretization.

#### Loading data from the Gene Expression Omnibus

The NCBI GEO is a large repository of gene expression and other profiling data, allowing public access to thousands of experiments. Given a GEO series ID, ChiBE retrieves the dataset and maps on the network, automatically. ChiBE displays the comparison (fold change) of the first two experiments in the dataset by default. Users can set the method that generates the displayed values from the loaded experiments. They can visualize a single experiment, or the average of a set of experiments, or compare two sets of experiments. Mapped values are color coded on the related molecules on the network, and values are displayed with tooltips.

### Managing biological complexity

Biological pathways are inherently complex and this reflects on the pathway models. One of our key design goals for ChiBE was to help users manage this complexity. ChiBE has a rich selection of tools for highlighting, viewing and editing (including zooming and scrolling), context sensitive property inspection, and interactive object move and resize.

Users can create new pathway views by selecting or highlighting a set of molecules or processes and cropping to that particular subnetwork in a new view to reduce complexity. Similarly, users may select or highlight parts of the network that they are not currently interested in and hide them. At any time during analysis, a view can be saved as a static image (in SVG, JPEG, BMP, or PNG file format) or in GraphML format for importing into another graph analysis tool.

The dynamic and interactive nature of ChiBE views requires pathways to be automatically drawn. A critical and unique component of ChiBE is the automatic layout algorithm supporting compound structures [27] used for visualizing molecular complexes and subcellular compartments. This algorithm was further customized for biological pathways to for example recognize and de-emphasize ubiquitously present small molecules such as ATP, nest subcellular compartments to represent biological containment relationships, and tile members of a molecular complex for more compact drawings.

**Figure 6:**
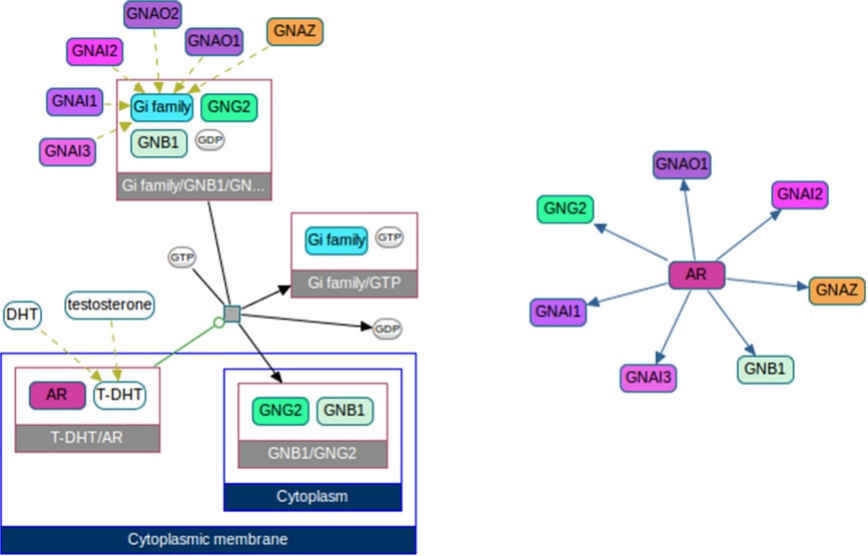
**SIF views with ChiBE.** A process diagram view (left) and its SIF view (right), showing controls-state-change relations.

To further reduce the complexity of large networks and complex pathways, it can be useful to visualize them as a simpler network. ChiBE can reduce a rich BioPAX pathway representation to a binary network between entities using a carefully selected set of rules [28] and display the network as a newly created view (Figure 6). Supported interaction types for Level 3 models include "controls-state-change-of", "controls-transport-of", "controls-expression-of", "controls-degradation-of", "catalysis-precedes", "in-complex-with", and "neighbor-of". These networks can be saved on disk in the Simple Interaction Format (SIF) for later use. A paths-between query can be performed on a SIF file, providing a set of genes of interest, through which one can obtain a connected subnetwork including these genes.

### ChiBE pathway representation

**Figure 7:**
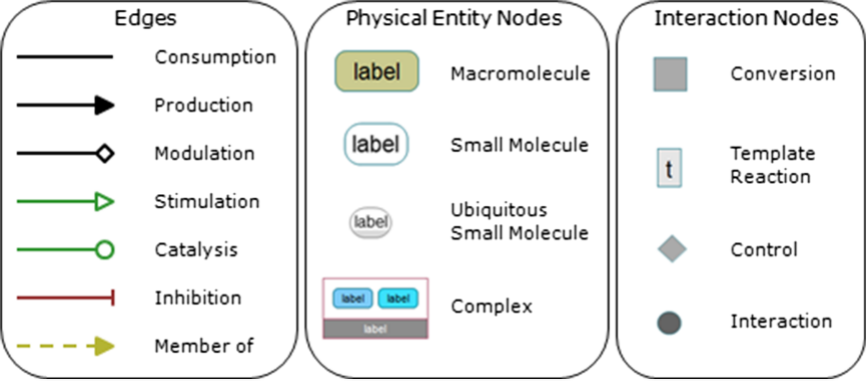
**Notation.** Notation for process description diagrams in ChiBE.

ChiBE pathway representation mostly complies with SBGN Process Description notation [20]. There are, however, a few differences, mostly due to the specific BioPAX semantics that have not been yet addressed in SBGN-PD or conflicts with other requirements. In several other instances, we simply chose to optimize user experience at the cost of relatively minor conflicts with the standard (Figure 7). We are working with the SBGN community to resolve these issues and expect to be fully compliant in the future. The differences are itemized below.

- **Control on controls:** In SBGN-PD, the target of an effector arc can only be a process node, but BioPAX allows defining controls on other controls. To address this difference, ChiBE uses a special node type for showing the controlled control, and directs the effector edge to the control node.
- **Input and output ports:** SBGN-PD uses two ports for collecting a process node’s input and output arcs. This feature allows unambigious drawing of reversible reactions, but as the graph gets more complex and node degrees increase, ports bring additional challenges to the already difficult problem of layout. Another side effect of the aforementioned mismatch in representing controls is that it is hard to draw reversible reactions for some cases. We opt to always draw reversible reactions as two reactions in opposite directions. As this choice also removes the need for ports, ChiBE does not use ports, drawing input and output arcs directly to the process node.
- **Generic relations:** SBGN-PD does not support abstractions such as homology relations and generic entities. However, these are defined in BioPAX, and they are frequently used in Pathway Commons. ChiBE draws a distinct arc from the generic molecule to its members.
- **Small molecule shape:** SBGN-PD uses a circle glyph for small molecules. The constant aspect ratio of the circle, however, causes problems in placing labels, especially for long small molecule names. ChiBE uses a single rounded rectangle glyph for all entities and differentiates small molecules by a white background.

### Comparison of ChiBE version 2 to related tools

The first version of ChiBE [10] supports BioPAX models of Level 2, experiment data overlay of tab delimited data, and basic querying of an older version of the Pathway Commons database. In version 2, we added support for BioPAX models of Level 3 and modified the graphical notation for better compliance with SBGN-PD. We also developed advanced graph-based querying for both the new version of the Pathway Commons database and for local models. In addition, integration to the cBioPortal and the GEO for easy access to genomic profiles have been made. Without this streamlining, finding and retrieving pathways, downloading and re-formatting data as well as often having to perform ID mapping take considerable effort and time for a user. Furthermore, ChiBE provides key technologies including BioPAX input and output, full support for visualizing compound structures such as molecular complexes and cellular locations in SBGN, and customized pathway layout to present this information to the user in an intuitive manner. Thus, ChiBE removes technical barriers to accessing popular network and profiling databases, making it a unique software tool for molecular biology and genomic researchers.

CellDesigner [29] is an excellent visual pathway editor, comparable to ChiBE in its capacity to display rich pathway information. It is, however, designed primarily for pathway curation and simulation of SBML models. It currently cannot import BioPAX models, nor can it dynamically perform graph-based searches and visually present the results. PathCase [30] has similar searching capabilities to ChiBE but is focused on metabolic pathways, and cannot overlay genomic or other types of profiling data. OmicsAnalyzer [31], a Cytoscape plugin, supports visualization of omics data in a network context but because its developers did not assume a standard network structure or a standard omics data structure, mapping between the data and the associated network is left to the user, which is prohibitively complicated in most cases. The tool is also limited to a simple interaction view, and is not capable of representing the rich process views that ChiBE can. Ingenuity Systems makes a commercial software system called IPA, which has similar dynamic searching, data overlay, and analysis workflow capabilities to ChiBE’s but it uses a proprietary simple interaction network representation as opposed to the rich public pathway information that ChiBE is based on. IPA also does not give users as much control over how queries are performed as ChiBE does. We believe that using a public, standard, and feature-rich pathway representation will be increasingly important as the public pathway corpus [4] and our capacity to use it to answer biological questions [32] grows. There are several other tools and Cytoscape plugins [33–36] that have portions of ChiBE’s functionality, but none provides a completely integrated workflow comparable to ChiBE.

## Conclusions

ChiBE allows users to tap into the detailed pathway information corpus for genomic data analysis. This would not be possible without its three key features: process views, specialized graph queries, and integration with genomic data repositories. Process views show the mechanism of events, bringing causality and context to the gene interactions. Graph queries enable users to define context specific boundaries for pathways. Automated mapping from genomic data repositories onto pathways streamlines the analysis, eliminating manual formatting steps. As demonstrated by the use cases, ChiBE’s uniquely powerful visualization and analysis workflow opens up new opportunities for scientific discovery.

## Availability and requirements

-  **Project name:** ChiBE
-  **Project home page:**http://code.google.com/p/chibe
-  **Operating system(s):** Platform independent
-  **Programming language:** Java
-  **Other requirements:** JRE 1.6 or higher
-  **License:** Eclipse Public License 1.0
-  **Any restrictions to use by non-academics:** None

## Abbreviations

BioPAX: Biological Pathways Exchange
ChiBE: Chisio BioPAX Editor
NCBI GEO: National Center for Biotechnology Information Gene Expression Omnibus
OWL: Web Ontology Language
SBGN-PD: Systems Biology Markup Language - Process Description
TCGA: The Cancer Genome Atlas.
